# Trends in overall survival and costs of multiple myeloma, 2000–2014

**DOI:** 10.1038/leu.2016.380

**Published:** 2017-01-20

**Authors:** R Fonseca, S Abouzaid, M Bonafede, Q Cai, K Parikh, L Cosler, P Richardson

**Affiliations:** 1 Mayo Clinic, Phoenix, AZ, USA; 2 Celgene Corporation, Summit, NJ, USA; 3 Health Economics and Outcomes Research, Truven Health Analytics Inc., an IBM company, Cambridge, MA, USA; 4 Binghamton University, Binghamton, NY, USA; 5 Dana Farber Cancer Institute, Boston, MA, USA

## Abstract

Little real-world evidence is available to describe the recent trends in treatment costs and outcomes for patients with multiple myeloma (MM). Using the Truven Health MarketScan Research Databases linked with social security administration death records, this study found that the percentage of MM patients using novel therapy continuously increased from 8.7% in 2000 to 61.3% in 2014. Compared with MM patients diagnosed in earlier years, those diagnosed after 2010 had higher rates of novel therapy use and better survival outcomes; patients diagnosed in 2012 were 1.25 times more likely to survive 2 years than those diagnosed in 2006. MM patients showed improved survival over the study period, with the 2-year survival gap between MM patients and matched controls decreasing at a rate of 3% per year. Total costs among MM patients have increased in all healthcare services over the years; however, the relative contribution of drug costs has remained fairly stable since 2009 despite new novel therapies coming to market. Findings from this study corroborate clinical data, suggesting a paradigm shift in MM treatment over the past decade that is associated with substantial survival gains. Future studies should focus on the impact on specific novel agents on patients’ outcomes.

## Key points

1. MM patient survival has increased steadily since 2000, mirroring the introduction of several novel therapies.
2. Costs among newly diagnosed MM patients have increased steadily since 2000, with a higher proportion of costs driven by the cost of inpatient admissions and outpatient services.

## Introduction

Multiple myeloma (MM)is a plasma cell neoplasm associated with its characteristic clinical complications: anemia, infections, renal impairment or bone destruction. MM is the second most commonly diagnosed hematological neoplasm, with an incidence rate of 6.2 per 100 000 individuals.^1^ In 2016, there were an estimated 30 330 newly diagnosed cases in the United States, accounting for ~1.8% of all new cancer cases.^2^ MM primarily affects the elderly and is typically diagnosed between the ages of 65–74 years, with estimated 5-year survival rate ranging from 34.5% to 49.6% from 2000 to 2008.^2^ Patients with MM experience severe bone, blood and renal complications, impacting therapy choices and quality of life.^3^ As MM is a progressive and mostly incurable disease,^2^ extending the time to disease progression in newly diagnosed patients is currently the primary treatment goal although cures may be attained in a small minority of patients.^4^

Traditional treatment regimens for the treatment of newly diagnosed MM disease date back to the 1960s, with melphalan plus prednisone as the first-line therapy along with combinations of vincristine, doxorubicin and dexamethasone.^5^ By the mid-1990s stem cell transplantation (SCTs) became common but primarily for younger patients with adequate kidney function.^6^ Over the past decades, one of the major advances in the treatment regimen of patients with MM has been the introduction of novel therapies, including immune-modifying drugs (thalidomide and lenalidomide) and proteasome inhibitors such as bortezomib.^5, 6, 7^ Many of these novel therapies, such as lenalidomide and bortezomib are currently recommended as initial treatment regimen in newly diagnosed patients irrespective of SCT eligibility.^3^ Phase III randomized trials have shown that addition of novel drugs such as bortezomib and lenalidomide to melphalan–prednisone improves both progression free (up to ~30 months) and median overall survival (~4.7 years).^8, 9^

In the last few years, the therapeutic landscape of MM improved even further because of the US FDA approvals of pomalidomide, another immune-modifying drug, in 2013, the monoclonal antibodies daratumumab and elotuzumab in 2015, as well as the new-generation proteasome inhibitors carfilzomib in 2012 and ixazomib in 2015.^10^ However, the addition of the novel therapies has raised logical concerns regarding the lifetime costs of MM treatment. Cost data from clinical studies typically revolved around specific interventions, smaller study populations and for a relatively short study period. There are few published real-world reports on patients with MM, and the available studies are limited to discrete assessments of few treatment trends^11^ and associated costs^12^ or survival.^13^ Despite evidence of improved survival and increased costs, little research has been conducted that comprehensively examines trends in MM outcomes over time, assessing both economic and clinical outcomes. This real-world study was designed to evaluate trends in MM treatment use, associated healthcare costs and patient survival in a large newly diagnosed MM patient population in the United States. Furthermore, this study also assessed the survival outcomes associated with novel therapies and percent of total healthcare costs attributable to the MM treatments.

## Materials and methods

### Study design and study patients

This retrospective, observational, matched controls study was conducted using US administrative claims data from the Truven Health Analytics MarketScan Commercial and Medicare Supplemental Databases. Patients with at least two non-diagnostic medical claims (that is, claims for a professional encounter, not solely for diagnostic or testing purpose such as laboratory, diagnostic radiology, imaging and so on) with MM (ICD-9 diagnosis code 203.0 ×) at least 30 days apart between 1 January 2000 and 30 September 2015 were included in the MM cohort. The date of the first diagnosis claim for MM was designated as index date. Patients with any claims for MM treatment (including autologous SCT) in the 12-month pre-index period were excluded.

Control patients were randomly selected from a pool of patients without any medical claims for an MM diagnosis but with a medical claim indicating any other diagnosis. The date of the first medical claim for any condition was designated as index date for controls. Controls were directly matched with MM cohort in a 1:1 ratio based on the following variables: index year, age (±5 years), gender and geographic region.

All patients were required to be 18 years and older on the index date and have at least 12 months of continuous enrollment with medical and pharmacy benefits prior to index and for at least 3 months post index.

### Data sources

Both the Truven Health Analytics MarketScan Commercial Claims and Encounters and Medicare Supplemental Databases provide detailed outcome measures including resource utilization and associated costs for healthcare services performed in both inpatient and outpatient settings for ~40 million individuals covered annually by a geographically diverse group of self-insured employers and private insurance plans across the US The commercial Database contains the pharmacy and medical claims of employees and their dependents, and the Medicare Supplemental database profiles the healthcare experience of individuals with Medicare supplemental insurance paid for by employers, capturing both the patient and health plan borne healthcare costs. The MarketScan Research Databases were further linked to the Social Security Administrations Master Death File to obtain patient survival data from 2006 to 2012. All study data were accessed with protocols compliant with US patient confidentiality requirements, including the Health Insurance Portability and Accountability Act of 1996 regulations. As the database is fully de-identified and compliant with the HIPAA; consequently, this study was exempted from Institutional Review Board approval.

### Outcomes

Patient demographics measured as of the index date included age at diagnosis, gender, insurance type, region of residence, total continuous enrollment post index and index year. Owing to the time period of the study, the novel treatments considered were bortezomib, carfilzomib, lenalidomide, panobinostat, pomalidomide and thalidomide. The non-novel treatments included arsenic trioxide, bendamustine, busulfan, cisplatin, cyclophosphamide, doxorubicin, etoposide, melphalan, prednisone, dexamethasone, rituximab, vincristine and vorinostat.

All-cause healthcare resource utilization and costs were evaluated by categories of inpatient admissions, outpatient services and prescription fills associated with any condition, including wellness visits. All-cause healthcare costs were defined as the sum of health plan paid and patient paid costs incurred from fully adjudicated medical services (including inpatient admissions and outpatient services) and prescription claims during the follow-up period. Total all-cause costs were attributed to costs from inpatient admissions, outpatient services and outpatient prescriptions. In addition, MM treatment-related drug costs consisted of prescription costs from outpatient pharmacy services defined using the National Drug Code and infusion costs from medical services associated with MM treatment defined using the Healthcare Common Procedure Coding System codes. Resource utilization and costs were reported as per patient per month (PPPM), calculated as medical service and prescription use and associated costs divided by number of days of enrollment and multiplied by 30 days.

Survival time was measured as time from index date (that is, diagnosis date for MM patients) to the date of death, even where death occurred after disenrollment from the MarketScan Research Databases; patients without a date of death were assumed alive at the end of the study period (30 September 2015). Survival estimates were assessed by types of treatment among newly diagnosed MM patients, and also compared between MM patients and matched controls over the study period. In addition, the proportion of patients who died or survived at least 2, 3 or 5 years after diagnosis were also described.

Not all identified MM patients were treated, likely representing cases of smoldering MM (SMM), but this is impossible to differentiate as there is no code specific to that stage of the disease. To assess the effect of the inclusion of these patients on the results, a subgroup analysis was conducted to examine the overall survival and total PPPM all-cause healthcare costs only among newly diagnosed MM patients who actually received MM treatment within 1 year after diagnosis.

### Statistical methods

Categorical variables were presented as the frequency and percentages and continuous variables were summarized by providing the means and s.d.s. To compare difference in study outcomes between MM patients and controls, *χ*^2^-tests were used for categorical variables and *t*-tests were for continuous variables. The Kaplan–Meier method of survival analysis was used to display overall survival, and log-rank tests were used to evaluate the difference in survival distributions of two groups. Differences were considered significant if the *P*-value was <0.05. All statistical analyses were conducted using SAS version 9.4 (SAS Institute Inc., Cary, NC, USA).

## Results

### Patient demographics

**Figure 1:**
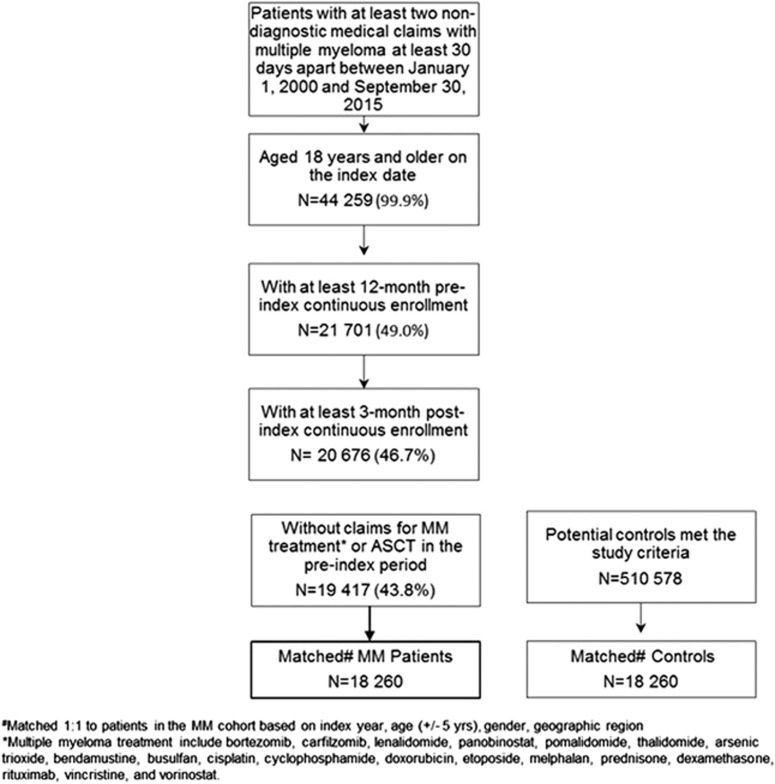
Patient selection.

A total of 19 417 newly diagnosed adult MM patients satisfied the study criteria prior to matching to controls; a pool of 510 578 potential control patients were identified for matching. Of those, 18 260 MM patients were directly matched to controls and included in the comparison analyses (Figure 1).

For the 19 417 newly diagnosed MM patients, the average age at diagnosis was 65.9 years and 44.9% were female, which was similar for the matched MM patient and controls (Table 1). Compared with patients treated with non-novel therapy, the relative age of MM patients treated with novel therapies within 1 year after diagnosis was different throughout the study period, whereas those receiving novel therapies were 3–4 years younger from 2004 to 2010, they were similar in age or older in other years. In addition, no significant difference in urban vs rural location was observed between patients treated with non-novel therapy and those who received novel therapy during the study period (*P*=0.89).

### Trends in MM treatment

**Figure 2:**
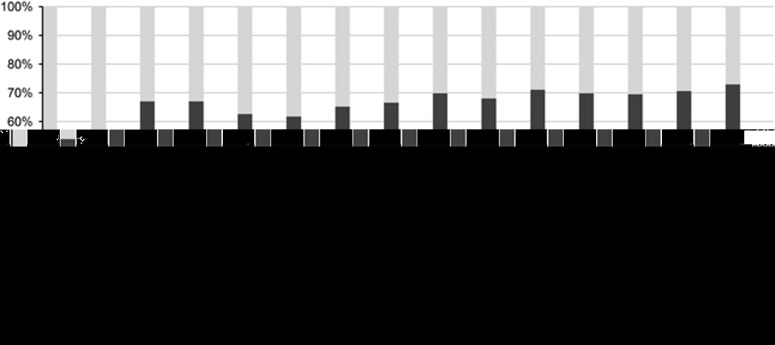
Multiple myeloma treatment used within 1 year after diagnosis, by year of diagnosis. Note: novel treatment include: bortezomib, carfilzomib, lenalidomide, panobinostat, pomalidomide and thalidomide. Non-novel treatment include: arsenic trioxide, bendamustine, busulfan, cisplatin, cyclophosphamide, doxorubicin, etoposide, melphalan, prednisone, dexamethasone, rituximab, vincristine and vorinostat.

MM treatment utilization was described among the entire newly diagnosed MM population (*n*=19 417) by identifying utilization of novel and non-novel pharmacotherapies within 1 year after diagnosis. The percentage of patients who used at least one novel and one non-novel therapy (for example, lenalidomide+dexamethasone) within 1 year after diagnosis increased across the study period from 7.5% in 2000 to 56.4% in 2014. As expected treatment with only novel therapies after diagnosis, such as bortezomib, lenalidomide or thalidomide, was initially rare but increased from 1.1% to 6.6% during the study period (Figure 2). Approximately 28% of patients with MM did not receive any treatment within 1 year after diagnosis, possibly indicating SMM or other conditions suggesting a poor candidate for therapy.

### Trends in overall survival

Overall, 7139 patients who were newly diagnosed with MM during 2006–2012 were able to link with the social security administration Master Death File. Patients younger than 65 years old at diagnosis had better survival than those 65 years and older (*P*<0.01). 19.5% of MM patients had a SCT during the same time period, with an improved survival experience than those without SCT (*P*<0.01). Furthermore, among these newly diagnosed patients who received a MM treatment (*n*=4902), patients treated with novel therapies within 1 year of diagnosis showed significantly better survival than those with only non-novel therapies (*P*=0.01).

**Figure 3:**
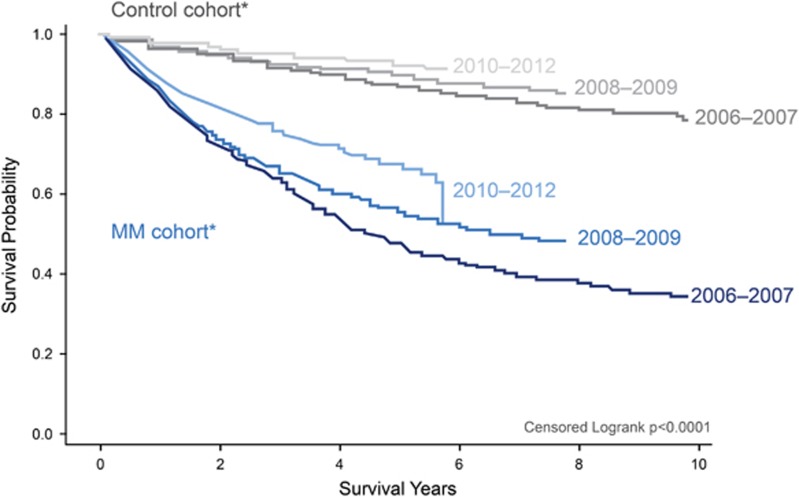
Survival estimates of matched MM patients and controls, by year of diagnosis. *Year ranges represent the year of diagnosis. Log-rank tests were used to evaluate the difference in survival distributions of two groups. Note: by linking to the SSA Master Death File, survival was measured as time from diagnosis date to the date of death obtained from the SSA, time from diagnosis date to the date of inpatient death, or time from diagnosis date to 30 September 2015; survival estimates were presented for multiple myeloma patients diagnosed and treated during 2006–2012 (*n*=9521).

**Figure 4:**
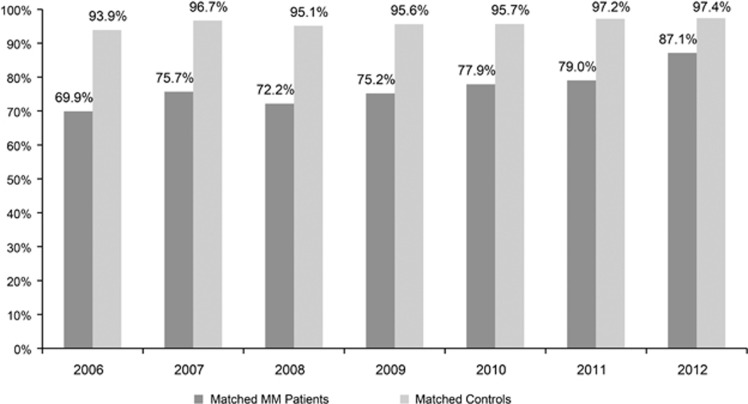
Percentage of patients who survived at least 2 years between matched MM patients and controls. Note: by linking to the SSA Master Death File, survival was measured as time from diagnosis date to the date of death obtained from the SSA, time from diagnosis date to the date of inpatient death, or time from diagnosis date to 30 September 2015; survival rates are presented for multiple myeloma patients diagnosed and treated during 2006–2012 (*n*=9521).

The survival disparity between MM patients and their matched controls decreased substantially over time (Figure 3). A greater proportion of MM patients survived for 2 years post diagnosis in 2012 (87.1%) than in 2006 (69.9%), whereas 2-year survival was fairly consistent for matched control patients (93.9–97.4% during 2006–2012; Figure 4).

### Trends in resource utilization and cost

**Figure 5:**
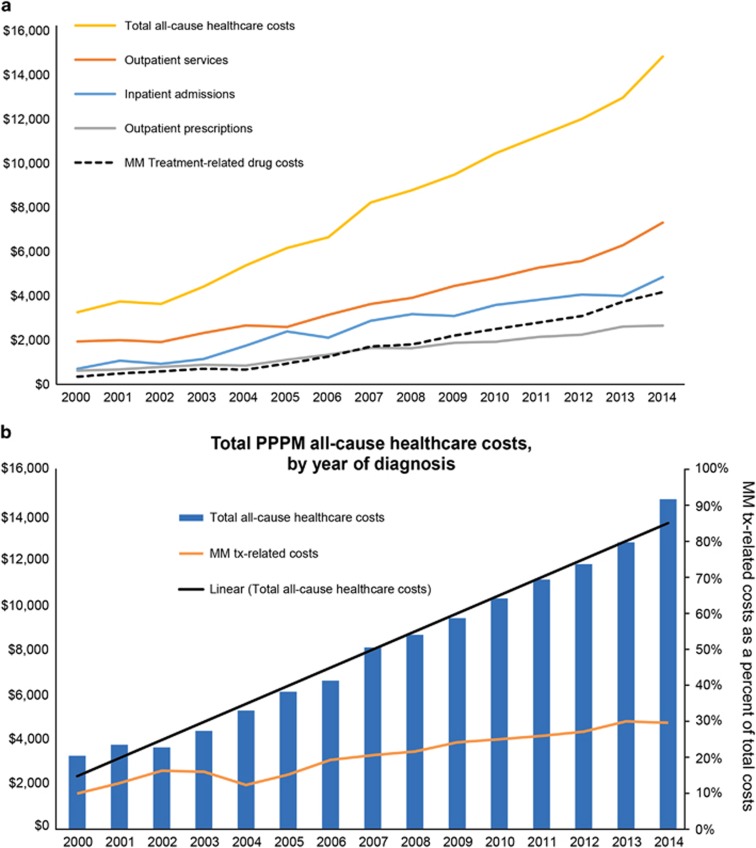
(**a**) Trends in PPPM total and component costs, by year of diagnosis. Note: costs are measured as PPPM and calculated as (costs/number of days of enrollment) × 30 days; outpatient services include emergency room, physician visits, laboratory, radiology and infusion administration services. (**b**) Total PPPM all-cause healthcare costs, by year of diagnosis. Note: costs are measured as PPPM and calculated as (costs/number of days of enrollment) × 30 days; MM treatment-related drug costs include outpatient pharmacy prescription costs (NDC codes) and injectable costs from outpatient services (HCPCS codes).

Total PPPM all-cause healthcare costs increased from $3263 PPPM in 2000 to $14 656 PPPM in 2014 among newly diagnosed MM patients, which were primarily driven by costs of outpatient services (Figure 5a). Costs attributable to outpatient services increased from $1945 to $7200 PPPM from 2000 to 2014, but its proportion of total healthcare costs decreased from 59.6% in 2000 to 49.1% in 2014. Outpatient services included a wide range of healthcare utilizations such as emergency room, physician visits, laboratory, radiology and infusion administration services. The PPPM rates of emergency room visits increased from 0.14 in 2000 to 0.90 in 2014, contributing to PPPM costs growing from $21 to $220 PPPM over the same time period.

In 2000, hospitalization costs accounted for 21.5% of total healthcare costs, increasing to 32.7% in 2014. The PPPM hospitalization rates steadily increased over the study period, from 0.045 in 2000 to 0.109 in 2014, with parallel increase in hospitalization costs from $701 to $4797 PPPM. Some of the increase in hospitalization costs is likely due to the increased use of SCT, which steadily increased from 0.027 per patient-year in 2000 to 0.090 in 2008 and 0.165 in 2014. In 2000, 4.3% of all inpatient stays among MM patients included SCT, which increased to 9.5% by 2014. Paralleling this increase in SCT incidence was a marked increase in SCT-related costs, which was from $124 PPPM in 2000 to $1612 PPPM in 2014.

Inpatient admissions for specific diagnoses and the associated costs were also assessed. The hospitalization rate for sepsis or infection-related hospitalization increased from 0.013 in 2000 to 0.038 PPPM in 2014, with associated costs increased from $209 to $1782 PPPM. Likewise, the hospitalization rate for anemia or neutropenia-related increased from 0.009 in 2000 to 0.070 PPPM in 2014 with associated costs increased from $145 to $3970 PPPM. The same trend was observed in the number of inpatient admissions due to pathologic fracture, from 0.002 in 2000 to 0.014 PPPM in 2014, with corresponding costs increased from $36 to $695 PPPM.

MM treatment-related drug costs, including both outpatient pharmacy prescriptions and office administered injectables, but excluding SCT, were described. As shown in Figure 5b, MM treatment-related drug costs increased from 2000 to 2014 at a rate similar to but slower than total all-cause costs. MM treatment-related drug costs accounted for 10.6% of total healthcare costs among MM patients in 2000 ($346 PPPM), increasing to 20.3% in 2007 and 28.5% in 2014 ($4179 PPPM). Healthcare costs were also compared between matched MM patients and their controls. Monthly all-cause costs increased for both cases and controls over the study period. Among matched MM patients, PPPM costs increased from $3263 in 2000 to $15 546 (476%) in 2014, compared with that in matched controls, which doubled from $686 to $1255 (182%) over the same time period.

### Subgroup analyses

To validate the robustness of the study findings, a subgroup analysis was performed only among newly diagnosed MM patients who actually received treatment within 1 year after diagnosis. Among these patients, the percentage of deaths decreased from 67.2% in 2006 to 21.4% in 2012 (a qualitatively higher decrease than we found in the total MM population); in addition, the percentage of patients who survived for 2 years post diagnosis increased from 69.8% in 2006 to 86.1% in 2012, similar to the findings from our primary analysis. The total PPPM all-cause healthcare costs increased from $4412 PPPM in 2000 to $18 424 PPPM in 2014 among patients with MM treatment; similar in trend to the main analysis, MM treatment-related drug costs increased slower than total all-cause costs during the study period, accounting for 9.4% of total healthcare costs in 2000 and 31.0% in 2014.

## Discussion

This study is one of the first to describe the treatment patterns, healthcare costs and overall survival among newly diagnosed patients with MM over a span of 15 years using a large administrative claims database that is expected to be representative of insured MM patients in the United States.^2^ Our study showed that the total PPPM all-cause healthcare costs were high for MM patients, which were mainly borne by the payer. The increase in total healthcare costs has become greatly unaffordable to patients with MM, their families and the society. The current study period spans the approval of several novel therapies in MM that have led to a changing treatment landscape over the last decade.^7^ The impact of novel therapies has resulted in a paradigm shift in the management of MM with substantially improved outcomes, measured by both disease-free and overall survivability of MM patients.^8, 9, 14, 15^ Our analysis corroborates these findings as overall survival was significantly improved across the index years. Importantly, patients receiving novel therapies had better survival outcomes compared with those who were managed with only non-novel drugs from 2000 to 2014, without an overt reason to believe that bias would favor the use of novel therapeutics by the treating providers (for example, healthier patients). This improvement was greater for patients diagnosed and treated after 2010 compared with those treated earlier.^10^ As expected, non-MM control patients had substantially better survival outcomes than MM patients treated, but the gap was significantly smaller for patients diagnosed later in the study period.

The economic impact of novel therapies has become a concern for cancer treatment in general^16^ and has been specifically highlighted in MM.^17, 18^ Although oncology treatment costs have increased, our findings also suggest a steady but greater increase in all-cause healthcare costs in other cost categories over the same time period. We found that the costs attributable to the MM treatment-related drugs increased slower than the costs of inpatient admission and outpatient services. In 2014, MM treatment-related drug costs accounted for ~30% of the total healthcare costs for MM patients, and increased only 5% from 2009 as a proportion of total costs despite new novel agents coming to market in this time period. Consistent with prior research documenting substantive costs associated with MM-related comorbidities, such as bone and skeletal complications,^19^ this study demonstrated that the increase in the total PPPM costs was driven, in part, by non-drug-related outpatient services and inpatient admissions. One possibility is that this is also a reflection of the improved longevity of patients with greater propensity to develop complications due to improved life expectancy.

Ideally, discussions around medication value, particularly in oncology, should take into account not only medication costs but also other related healthcare costs and outcomes improvement.^20, 21, 22^ Our analysis quantifies increases in MM treatment-related drug costs alongside a substantial increase in patient survival. Although still present, the survival gap between MM patients and their matched controls shrank considerably over the study period, suggesting that MM patients’ life expectancy is close to that of patients with other health problems. Where control patients were 1.34 times more likely to survive 2 years than their matched MM patients diagnosed in 2006, this difference was reduced to 1.12 times for those diagnosed in 2012. In other words, MM patients newly diagnosed in 2012 were 1.25 times more likely to survive 2 years than patients diagnosed in 2006. Likewise, compared with 2006, MM treatment-related drug costs increased by $2084 by 2012, whereas 2-year survival increased from 69.9% to 87.9%, translating to an additional $121 PPPM per additional 1% increase in 2-year survival. To better evaluate the value of new MM treatments, it is worthwhile to estimate life year gained using life expectancy or similar metrics and determine the incremental cost-effectiveness ratio in the future study, particularly when there is sufficient real-world data on the latest wave of new novel therapies. Given the recent advent of a number of new effective therapies in MM, this analysis may now be forthcoming in the near future.

Despite the strength of the real-world data and direct matching, this study had certain general limitations that are associated with such claim based observational studies. First, this study was limited to only those individuals with commercial health coverage or private medicare supplemental coverage. Consequently, results of this analysis may not be generalizable to patients with other insurance or without health insurance coverage. Second, the identification of MM population relied on diagnosis codes recorded on medical claims and are therefore limited by completeness and accuracy of medical coding. Third, this study may underestimate total healthcare costs of MM patients given that costs estimates only account for direct healthcare costs for services reimbursed by commercial insurers. Indirect costs and caregiver burden were not included because relevant data cannot be captured from the claims database. We calculated resource utilization and costs as PPPM with no inflation adjustment that would give a greater value to older dollars, which might give the appearance of flatter cost increases than were truly present. Finally, the analysis did not include patient’s race or ethnicity because relevant data cannot be captured from administrative claims. However, previous studies have reported variations in survival experience of MM patients by difference races;^23^ thereby, it is worthwhile to control this variation in matching. Similarly, this study could not control for MM disease severity, which is unlikely but may have possibly changed over the study period and could impact treatment patterns, costs and patient survival.

## Conclusion

Newly diagnosed MM patients diagnosed after 2010 have better survival outcomes than those diagnosed in earlier years. Total healthcare costs among patients with newly diagnosed MM have increased steadily since 2000; however, MM treatment-related drug costs increased slower than other cost components and remained a minority of total costs.

## Figures and Tables

**Table 1:** Demographic characteristics of study patients

*Characteristics*	*Newly diagnosed MM Patients (*N*=19 417)*		*Matched MM patients (*N*=18 260)*		*Matched controls (*N*=18 260)*	
Age at diagnosis, years, (mean, s.d.)	65.85	12.33	64.89	12.00	64.78	11.95
						
*Gender (*N*, %)*						
Male	10 704	55.1%	9795	53.6%	9795	53.6%
Female	8713	44.9%	8465	46.4%	8465	46.4%
						
*Geographic region (*N,*%)*						
Northeast	3264	16.8%	3097	17.0%	3097	17.0%
North Central	6145	31.6%	5538	30.3%	5538	30.3%
South	6592	33.9%	6332	34.7%	6332	34.7%
West	3327	17.1%	3211	17.6%	3211	17.6%
Unknown	89	0.5%	82	0.4%	82	0.4%
						
*Location (*N*, %)*						
Rural	2913	15.0%	3027	16.6%	2759	15.1%
Urban	16504	85.0%	15 233	83.4%	15 501	84.9%
